# Adipose Tissue Plasticity and Adipogenesis: From Cellular Mechanisms to Therapeutic Targets in Obesity

**DOI:** 10.1007/s13679-026-00733-4

**Published:** 2026-06-24

**Authors:** Sílvia Rocha, Carina Proença, Félix Carvalho, Eduarda Fernandes

**Affiliations:** 1 LAQV, REQUIMTE, Laboratory of Applied Chemistry, Department of Chemical Sciences, Faculty of Pharmacy, University of Porto, Rua de Jorge Viterbo Ferreira n. º 228, Porto, 4050-313 Portugal; 2 UCIBIO‑ Applied Molecular Biosciences Unit, Laboratory of Toxicology, Department of Biological Sciences, Faculty of Pharmacy, University of Porto, Porto, 4050-313 Portugal; 3 Associate Laboratory i4HB ‑ Institute for Health and Bioeconomy, Faculty of Pharmacy, University of Porto, Porto, 4050‑313 Portugal

**Keywords:** Adipogenesis, Adipose tissue plasticity, Obesity, Adipocyte differentiation, Metabolic homeostasis.

## Abstract

**Purpose of Review:**

This review provides an overview of adipose tissue plasticity and adipogenesis as central processes regulating adipose tissue expansion, remodeling, and metabolic function in health and obesity, highlighting their relevance as potential therapeutic targets.

**Recent Findings:**

Adipogenesis is a tightly regulated process involving the differentiation of adipose progenitor cells into mature adipocytes through coordinated transcriptional cascades, primarily driven by peroxisome proliferator–activated receptor (PPAR)γ and CCAAT/enhancer–binding protein (C/EBP) family members. This process is further modulated by multiple signaling pathways, including wingless–related integration site (Wnt), bone morphogenetic proteins (BMPs), and insulin signaling, which collectively regulate adipocyte differentiation and metabolic function. Under physiological conditions, the adipose tissue exhibits remarkable plasticity, with adipogenesis supporting lipid buffering capacity and tissue renewal. In obesity, however, chronic nutrient excess and hormonal dysregulation impair this process, favoring adipocyte hypertrophy, hypoxia, and chronic inflammation. These alterations disrupt adipokine secretion and promote ectopic lipid deposition, thereby contributing to metabolic disorders, including insulin resistance and cardiometabolic disease.

**Summary:**

Adipose tissue plasticity and adipogenesis are critical determinants of metabolic health. Dysregulation of these processes underlies adipose tissue dysfunction and contributes to the development of obesity-related comorbidities. Targeting adipogenesis and promoting healthy adipose tissue remodeling represent promising strategies for restoring metabolic homeostasis and mitigating obesity-associated diseases.

## Introduction

Obesity is a complex and multifactorial disease characterized by excessive fat accumulation that compromises metabolic health. Its development results from a dynamic interplay between genetic susceptibility, environmental influences, lifestyle factors, and endocrine and metabolic regulation [1, 2]. Although traditionally attributed to an imbalance between energy intake and expenditure, current evidence indicates that obesity is not solely a consequence of energy excess but also reflects alterations in the capacity of adipose tissue to expand and adapt to metabolic demands [3].

Adipose tissue plays a central role in this process due to its remarkable capacity to remodel in response to nutritional and hormonal cues. This adaptability, commonly referred to as adipose tissue plasticity, encompasses coordinated structural, cellular, and functional changes that enable the tissue to maintain metabolic homeostasis [4]. These adaptations involve changes in adipocyte size, number, and phenotype, allowing adipose tissue to respond dynamically to fluctuations in energy balance and environmental signals [3].

Under physiological conditions, adipose tissue expansion occurs through a balance between adipocyte hypertrophy (enlargement of existing adipocytes) and hyperplasia (formation of new adipocytes from progenitor cells). This coordinated response enables efficient lipid storage while preserving insulin sensitivity and preventing ectopic lipid accumulation. In this context, hyperplasia is generally considered a protective mechanism, as it supports the generation of smaller, metabolically functional adipocytes [5].

Adipogenesis, the process by which adipose progenitor cells differentiate into mature adipocytes, is a key determinant of this adaptive capacity. It ensures the generation of new, metabolically competent adipocytes, thereby supporting healthy tissue expansion and long-term energy storage [6]. This process is tightly controlled by transcriptional networks and signaling pathways that regulate cell commitment, differentiation, and functional maturation. When properly regulated, adipogenesis contributes to metabolic flexibility by enhancing lipid buffering capacity and maintaining endocrine balance [7].

In contrast, impaired adipogenesis is a hallmark of dysfunctional adipose tissue in obesity. Reduced adipogenic potential, together with persistent inflammatory and metabolic stress, favors adipocyte hypertrophy over hyperplasia [8]. This dysfunctional expansion is associated with hypoxia, fibrosis, and altered adipokine secretion, ultimately leading to ectopic lipid accumulation and systemic metabolic disturbances, including insulin resistance and cardiometabolic disease [9].

In addition to adipocyte formation, adipose tissue retains the ability to dynamically adapt its phenotype through processes such as transdifferentiation and browning, further highlighting its plastic nature. These adaptive mechanisms, regulated by environmental and molecular cues, expand the functional repertoire of adipose tissue and represent potential targets for therapeutic intervention [10].

Given the central role of adipogenesis and adipose tissue plasticity in determining metabolic outcomes, a deeper understanding of the underlying cellular and molecular mechanisms is essential. This review specifically focuses on the molecular regulation of adipogenesis and its integration with adipose tissue plasticity, highlighting transcriptional, signaling, and epigenetic mechanisms that govern adipocyte differentiation and function, as well as their therapeutic implications.

## Adipogenesis and Adipocyte Differentiation

The progressive expansion and remodeling of adipose tissue in response to nutritional demands ultimately converge on a tightly controlled biological process: adipogenesis. Defined as the differentiation of precursor cells into mature adipocytes capable of storing lipids and exerting endocrine functions, adipogenesis is a highly orchestrated cascade that is critical for maintaining adipose tissue homeostasis [11]. This process occurs within specialized adipose tissue depots that are functionally and metabolically distinct, and the health of adipose tissue depends on its ability to expand in a well–regulated state and safely store excess energy.

Under physiological conditions, efficient and tightly regulated adipogenesis enables the expansion of subcutaneous adipose tissue (SAT) and maintains adipose tissue homeostasis by supporting hyperplastic growth, preserving insulin sensitivity, and preventing ectopic lipid deposition, thereby protecting non–adipose organs from lipotoxic injury. Conversely, impaired adipogenesis disrupts these protective mechanisms by compromising the molecular cascade responsible for adipocyte differentiation and favoring hypertrophic, inflamed, and metabolically rigid adipose depots. Understanding these processes is therefore essential to contextualize adipose tissue plasticity and its systemic metabolic consequences in obesity.

Classically, adipogenesis unfolds through distinct but interdependent stages, including lineage commitment, early and intermediate differentiation, and terminal maturation. Each of these stages is governed by specific cellular, hormonal, and transcriptional regulatory mechanisms.

### Cellular Origin and Lineage Commitment

Adipogenesis begins within the stromal vascular fraction (SVF) of adipose tissue, where mesenchymal stem cells (MSCs) and stromal progenitors gradually restrict their multipotency and become increasingly biased toward an adipocyte lineage rather than alternative differentiation programs such as osteogenesis (bone), myogenesis (muscle), and chondrogenesis (cartilage), a stage referred to as early commitment. As lineage restriction progresses, these progenitors transition into pre–adipocytes, fibroblast–like cells that retain proliferative capacity but are primed to initiate a coordinated, multistep differentiation program driven by hormonal cues and a core adipogenic transcriptional cascade [11, 12].

The early commitment phase is controlled by transcriptional regulators such as zinc finger protein (Zfp)423 and early B-cell factor (Ebf)1 and Ebf2, which establish adipogenic competence and distinguish adipocyte–fated progenitors from other mesenchymal lineages. These factors modulate chromatin accessibility, creating a transcriptional environment that enables subsequent activation of the core adipogenic program, thereby defining the earliest molecular signature of adipocyte commitment [13, 14].

Once adipogenic competence is established, adipocyte–fated progenitors are specified but do not yet distinguish between white and brown adipocyte lineages. Several studies have demonstrated that most white adipocytes derive predominantly from mesenchymal precursor cells of an adipogenic lineage lacking myogenic factor (Myf)5 expression (Myf5^−^), indicating a developmental pathway distinct from the myogenic program. These Myf5^−^ progenitors differentiate into pre–adipocytes and subsequently mature adipocytes under appropriate endocrine and nutritional conditions [15–17]. In contrast, classical brown adipocytes originate from a Myf5^+^ lineage shared with skeletal muscle progenitors, reflecting their closer developmental relationship with myogenic tissues [16]. This developmental dichotomy underlies the distinct metabolic and thermogenic functions of white adipose tissue (WAT) and brown adipose tissue (BAT).

**Fig. 1:**
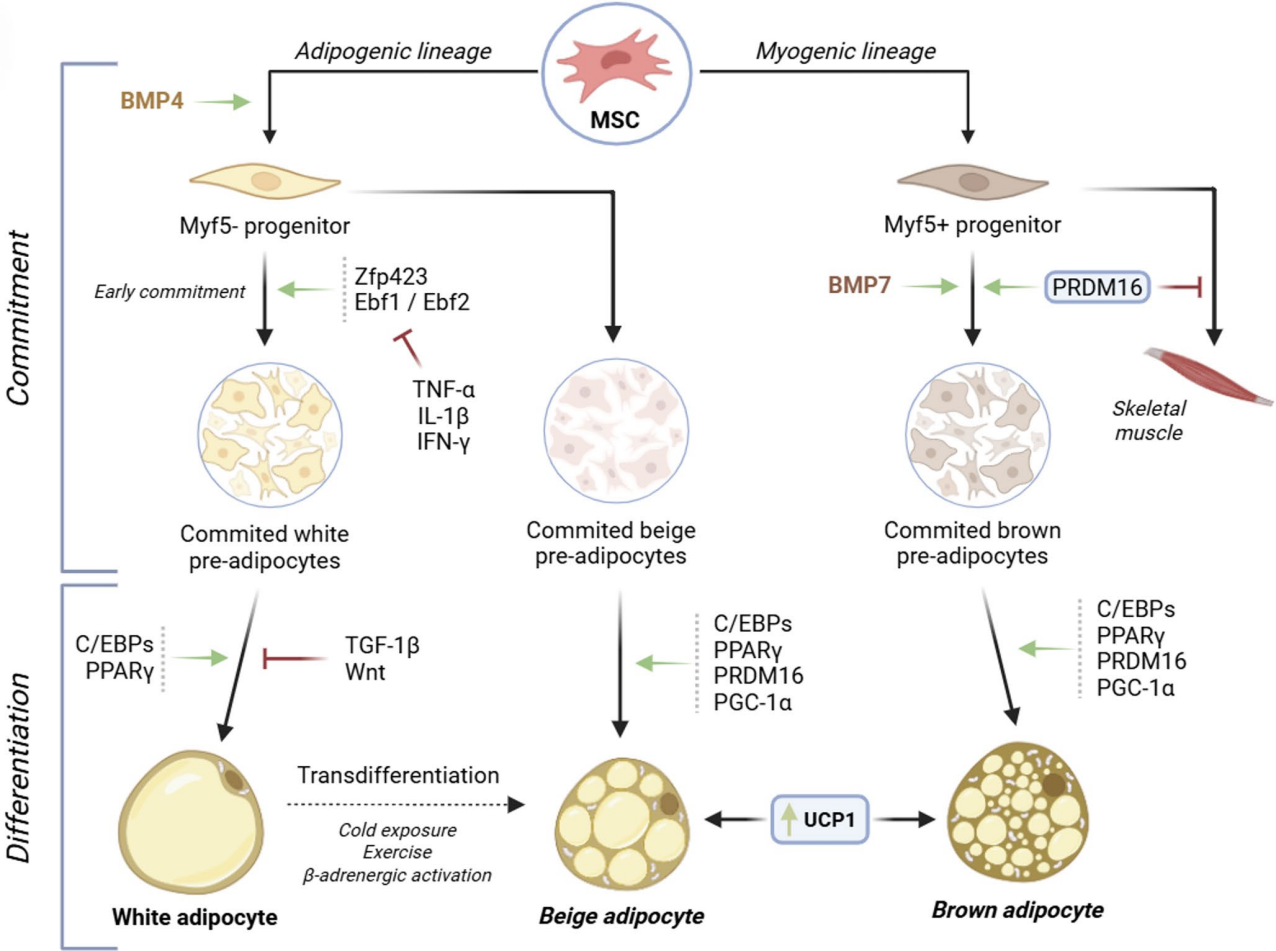
Adipocyte lineage commitment and differentiation. Mesenchymal stem cells (MSC) differentiate into adipogenic and myogenic lineages. Within the adipogenic lineage, Myf5⁻ progenitors commit to white or beige pre-adipocytes through zinc finger protein (Zfp)423 and B-cell factor(Ebf)1 and Ebf2, while inflammatory cytokines impair adipogenic commitment. White adipocytes differentiate via CCAAT/enhancer–binding protein (C/EBP) and peroxisome proliferator–activated receptor (PPAR)γ signaling and can transdifferentiate into beige adipocytes in response to cold exposure, exercise, or β-adrenergic activation. In parallel, Myf5⁺ progenitors give rise to brown adipocytes under bone morphogenic proteins (BMP)7- and PR domain–containing protein 16 (PRDM16)-dependent cues. Beige and brown adipocytes are characterized by increased uncoupling protein 1 (UCP1) expression and thermogenic capacity. Created in https://BioRender.com

In addition to white and classical brown adipocytes, beige adipocytes represent a third inducible thermogenic population. These cells originate from Myf5^−^ precursors within white adipose depots and can also emerge through the recruitment or transdifferentiation of mature white adipocytes in response to specific stimuli (Fig. 1) [18].

Other regulators such as T–box (TBX)15, PR domain–containing protein 16 (PRDM16, particularly relevant for beige adipocyte differentiation), and specific homeobox (HOX) gene clusters further contribute to depot–specific adipocyte identity. Subcutaneous progenitors exhibit higher HOXA5 expression, which is associated with a more insulin–sensitive phenotype, whereas visceral progenitors preferentially express HOXC9, a profile linked to increased inflammatory potential. In parallel with these molecular programs, adipocyte precursor cells are also heterogeneous across depots, with SAT enriched in adipogenic progenitors, while visceral adipose tissue (VAT) contains a higher proportion of fibro–inflammatory precursors with reduced differentiation capacity [19, 20]. These intrinsic molecular and cellular differences help explain the distinct metabolic behaviors of SAT and VAT and their differential susceptibility to adipose tissue dysfunction.

During the progression toward lineage commitment, pre–adipocytes gradually lose multipotency and adopt a fibroblast–like morphology. At this stage, pre–adipocytes express characteristic surface markers such as platelet–derived growth factor receptor (PDGFR)α, cluster of differentiation (CD)34, and CD29. The commitment phase is also highly sensitive to inflammatory cues, including tumor necrosis factor (TNF)–α, interleukin (IL)–1β, and interferon (IFN)–γ, which suppress early adipogenic regulators such as Zfp423 and members of the Ebf family. Persistent inflammatory signaling impairs adipocyte recruitment and favors hypertrophic expansion of existing adipocytes (Fig. 1) [21, 22].

Beyond cytokine-mediated effects, immune–stromal interactions strongly influence adipose progenitor fate and tissue remodeling. Adipose tissue macrophages (ATM), the predominant immune population within WAT, exert important regulatory effects on adipogenesis. Under lean conditions, alternatively activated M2-like ATMs support progenitor proliferation and adipogenic competence through anti-inflammatory IL-10 and transforming growth factor (TGF)-β, thereby promoting healthy hyperplastic expansion. In contrast, obesity promotes the accumulation of pro-inflammatory M1-like ATMs and lipid-laden metabolically activated ATMs, which secrete inflammatory mediators that suppress early adipogenic regulators such as Zfp423 and Ebf family members, impairing adipogenic commitment and shifting depot expansion toward pathological hypertrophy [23].

Adaptive immune cells also contribute to the regulation of progenitor cells. Regulatory T cells (Tregs) and invariant natural killer T (iNKT) cells maintain an anti-inflammatory microenvironment that preserves progenitor adipogenic potential and supports metabolic homeostasis. Their reduction in obesity amplifies inflammatory signaling, decreases hyperplastic capacity, and promotes extracellular matrix (ECM) deposition. Conversely, Th1-polarized T cell responses enhance ATM activation and fibrosis, further restricting adipose expandability [24]. In parallel, innate lymphoid cells (ILC)2 also modulate adipose plasticity by producing IL-5 and IL-13, which sustain eosinophil recruitment and M2 polarization, indirectly promoting beige adipocyte recruitment and thermogenic remodeling. Loss of ILC2 activity in obesity impairs this thermogenic axis and reduces progenitor response to browning stimuli, contributing to depot-specific dysfunction [25].

Together, these immune–stromal interactions integrate inflammatory tone, progenitor fate decisions, and adipose tissue plasticity, highlighting immune–metabolic crosstalk as a central regulatory axis in obesity-associated adipose dysfunction [26].

While the classical Myf5⁺/Myf5⁻ developmental framework has been fundamental for establishing the foundations of adipocyte lineage relationships, recent advances in single-cell RNA sequencing and spatial transcriptomics have considerably expanded this view [27]. Emerging evidence demonstrates that adipose progenitor populations are highly heterogeneous, comprising functionally distinct subsets with divergent adipogenic, fibrotic, inflammatory, and thermogenic potentials. These studies also revealed substantial lineage plasticity and strong microenvironmental influences on progenitor fate determination, supporting a more dynamic, plastic, and spatially organized model of adipose tissue development and remodeling [28].

These approaches revealed that adipose progenitors are far from a homogeneous population but instead comprise multiple specialized subsets, including DPP4⁺ interstitial progenitors with high self-renewal capacity, ICAM1⁺ committed pre-adipocytes with strong adipogenic potential, PDGFRβ⁺/Ly6C⁺ fibro-inflammatory progenitors, and beige-competent thermogenic precursors [27–29]. Spatial mapping further demonstrated that these populations occupy discrete micro-niches shaped by vascular proximity, ECM composition, and local inflammatory cues [30].

Together, these findings shift the current understanding from a binary Myf5⁺/Myf5⁻ model toward a more dynamic, heterogeneous, and spatially organized view of adipose tissue development. Rather than representing a uniform differentiation process, adipogenesis is now understood as a context-dependent and depot-specific phenomenon shaped by progenitor diversity, local inflammatory signals, ECM composition, and metabolic status. This refined framework helps explain depot-specific differences in adipogenic capacity and provides mechanistic insight into how microenvironmental signals influence progenitor fate, a concept that directly links lineage heterogeneity to the signaling pathways governing adipogenesis. Importantly, this conceptual shift also offers a more comprehensive framework for understanding adipose tissue dysfunction and pathological remodeling in obesity.

### Adipogenesis Signaling Pathways

The initiation and progression of adipogenesis are determined by a complex interplay of extracellular and intracellular signaling pathways that regulate the transition from lineage commitment to terminal differentiation. These pathways include hormonal, nutritional, and inflammatory cues that collectively determine whether adipocyte progenitors enter, sustain, or inhibit the adipogenic program. Broadly, adipogenic signaling pathways can be categorized into pro– and anti–adipogenic, reflecting their capacity either to promote activation of the core adipogenic transcriptional cascade or to restrain differentiation and maintain progenitors in an undifferentiated or alternative lineage state.

#### Pro–Adipogenic Pathways

Pro–adipogenic pathways comprise a set of signaling cascades that actively promote adipocyte differentiation by inducing the transcriptional machinery required for adipogenesis and coordinating the metabolic remodeling necessary for the development of mature adipocytes. Although multiple signals can promote adipogenic differentiation under specific physiological or experimental contexts, only a limited number of pathways are consistently recognized as central drivers of the adipogenic program, operating from lineage commitment to terminal maturation.

A defining early event in the adipogenic program is lineage commitment, which is primarily driven by bone morphogenic protein (BMP) signaling, particularly BMP4 and BMP7. BMP4 phosphorylates suppressor of mother against decapentaplegic (SMAD)1/5/8 and establishes a transcriptional state permissive for adipogenic differentiation, directing multipotent stromal cells away from osteogenic or myogenic programs and toward the adipocyte lineage [31]. In contrast, BMP7 selectively promotes brown adipocyte differentiation, inducing mitochondrial biogenesis, uncoupling protein 1 (UCP1) expression, and thermogenic programming via SMAD and p38 mitogen-activated protein kinase (MAPK) pathways. This commitment phase sets the foundation upon which subsequent differentiation signals act, ensuring that progenitor cells acquire the transcriptional and chromatin landscape necessary to respond effectively to pro–adipogenic hormonal stimuli [32]. Thus, BMP signaling functions as a key early determinant of adipocyte identity, preceding and enabling the initiation of the adipogenic transcriptional cascade (Fig. 1).

Once commitment is established, the early induction of adipogenesis strongly depends on cyclic adenosine monophosphate (cAMP)/protein kinase A (PKA) signaling. Elevated intracellular cAMP activates PKA, which phosphorylates the cAMP response element–binding protein (CREB), leading to its activation and subsequent binding to DNA sequences that regulate early adipogenic gene transcription, most notably CCAAT/enhancer–binding protein (C/EBP)β, the earliest transcriptional activator of the adipogenic program [11]. This early signal also facilitates mitotic clonal expansion, coupling cell proliferation with transcriptional priming in preparation for terminal adipocyte differentiation [33].

The progression from transcriptional initiation to mature adipocyte identity is driven by the insulin–like growth factor (IGF)–1/phosphoinositide 3–kinase (PI3K)/protein kinase B (Akt) pathway, which integrates hormonal and metabolic inputs to sustain adipogenesis. IGF–1 binds to its receptor, activating insulin receptor substrate (IRS) proteins that recruit and activate PI3K, leading to the production of phosphatidylinositol (3,4,5)–trisphosphate (PIP3), a critical step enabling full Akt activation [34]. Activated Akt enhances glucose uptake, stimulates lipogenic enzyme expression, inhibits anti–adipogenic regulators, and stabilizes peroxisome proliferator–activated receptor (PPAR)γ, consolidating the pro–adipogenic transcriptional program. Inhibition of PI3K or Akt blocks differentiation even after early transcriptional events have occurred, underscoring the indispensable anabolic role of this pathway [35].

Downstream of Akt, mammalian target of rapamycin complex (mTORC)1 operates as a master regulator of metabolic remodeling during adipocyte maturation. mTORC1 promotes protein synthesis, de novo lipogenesis, and organelle biogenesis, in part by activating sterol regulatory element-binding protein (SREBP)1c and other lipogenic transcriptional factors. This ensures that differentiating adipocytes acquire the metabolic capacity for triacylglycerides (TGs) synthesis, lipid droplet expansion, and endocrine functionality. Inhibition of mTORC1 markedly impairs lipid accumulation and suppresses the expression of late adipogenic genes, confirming its essential role in terminal differentiation [36].

Collectively, pro–adipogenic pathways create a hierarchical and cooperative network. BMP signaling establishes lineage commitment, cAMP/PKA/CREB initiates the transcriptional cascade, IGF–1/PI3K/Akt stabilizes and amplifies adipogenic identity, and mTORC1 drives the metabolic maturation required for functional adipocyte formation. These pathways ensure the coordinated acquisition of both transcriptional and metabolic features of the mature adipocyte.

#### Anti–Adipogenic Pathways

Anti–adipogenic pathways include signaling mechanisms that oppose adipocyte differentiation by inhibiting lineage commitment, blocking the activation of adipogenic transcription factors, or impairing the metabolic remodeling required for terminal differentiation.

Among all inhibitory regulators, adenosine monophosphate activated protein kinase (AMPK) is the most prominent. Acting as a master sensor of cellular energy stress, AMPK is activated under conditions of low adenosine triphosphate (ATP) availability and shifts metabolism toward catabolic processes. Through inhibition of mTORC1, suppression of lipogenic enzymes, phosphorylation–mediated suppression of PPARγ, and downregulation of C/EBPα, AMPK counteracts the anabolic and transcriptional requirements for differentiation. Its anti–adipogenic effects also extend to lipid metabolism, promoting fatty acid oxidation and reducing TGs synthesis, directly opposing the metabolic profile of mature adipocytes [36].

In addition to AMPK, the MAPK family, namely extracellular signal–regulated kinase (ERK), c-Jun N-terminal kinase (JNK), and p38 MAPK, acts as a central mediator linking extracellular cues to nuclear transcriptional programs. In obesity, MAPK cascades regulate appetite, adipocyte differentiation, thermogenic capacity, and inflammatory processes, acting both as pro–adipogenic (ERK) and anti–adipogenic (JNK and p38 MAPK) [37].

In the central nervous system, ERK1/2 activation enhances anorexigenic signaling by increasing the expression of pro–opiomelanocortin (POMC)–derived neuropeptides, thereby promoting satiety [38]. In contrast, within adipose tissue, MAPK signaling exhibits significant tissue– and time–specificity. Transient early ERK1/2 activation provides essential support during the induction phase of adipogenesis. Short–lived ERK signaling enhances C/EBPβ expression and facilitates mitotic clonal expansion, thereby contributing to the successful initiation of the adipogenic program [39].

Nevertheless, sustained or chronic ERK activity suppresses adipogenesis by inhibiting PPARγ, demonstrating that MAPK pathways can either promote or restrain adipocyte formation depending on cellular and temporal context. This temporal dichotomy is further illustrated by the requirement of ERK1 for terminal adipocyte differentiation, while prolonged MAPK activation remains inhibitory, highlighting the need for precisely timed ERK signaling throughout the adipogenic process [39, 40].

On the other hand, JNK and p38 MAPK play central roles in obesity–associated inflammation signaling. Their activation in adipocytes and resident ATM stimulates the production of pro–inflammatory cytokines that disrupt insulin receptor signaling, thereby exacerbating insulin resistance. These inflammatory responses establish MAPK signaling as a critical contributor to metabolic deterioration and impaired adipose tissue remodeling [41].

The TGF–β superfamily, particularly TGF–β1 and activins, also acts as a potent inhibitory signaling pathway in adipogenesis. Unlike BMPs, which promote adipogenic commitment, TGF–β1 signals through SMAD2/3 to repress PPARγ expression and maintain progenitor cells in an undifferentiated state. TGF–β signaling disrupts early adipogenic induction and interferes with C/EBPβ activation, effectively blocking the initiation of the adipogenic cascade [42]. In vivo, elevated TGF–β1 levels are associated with fibrosis and reduced adipose tissue expandability, further underscoring its inhibitory role (Fig. 1) [43].

Additionally, canonical wingless–related integration site (Wnt)/β–catenin exerts strong anti–adipogenic effects. Wnt activation stabilizes β–catenin and prevents the expression of PPARγ and C/EBPα, thereby maintaining progenitors in a pre–adipogenic or fibroblastic state (Fig. 1). While inhibition of adipogenesis may limit adipose expansion, chronic Wnt activation restricts the formation of beige adipocytes, impairs tissue remodeling, and promotes hypertrophic and dysfunctional adipocytes [44]. Wnt signaling also regulates incretin secretion in the gastrointestinal tract, linking this pathway to systemic glucose metabolism [45]. Notch signaling similarly restricts adipogenic differentiation by inducing Hes/Hey transcriptional repressors that interfere with adipogenic gene networks. Although sustaining progenitor proliferation, experimental studies showed that Notch signaling inhibits the cell–cycle exit required for terminal differentiation, leading to the loss of C/EBPα and PPARγ induction [46].

In parallel with canonical anti–adipogenic signaling pathways, endocrine regulators arising from the gastrointestinal tract also influence adipose tissue remodeling and systemic energy balance. Among these, glucagon–like peptide (GLP)–1 acts primarily through the gut-brain axis to suppress appetite, reduce calorie intake, and improve metabolic homeostasis. In addition to these central effects, GLP–1 receptor agonists have been associated with indirect modulation of adipose tissue function, including improved insulin sensitivity, activation of AMPK–related pathways, reduced inflammation, and increased thermogenic potential [47, 48]. However, the extent to which GLP–1 exerts direct anti-adipogenic effects in adipocytes remains incompletely understood and is still under debate. Thus, GLP-1 is best understood as an endocrine regulator that indirectly affects adipose tissue expandability rather than a canonical intracellular anti-adipogenic pathway.

In addition to inflammatory and hormonal regulation, mechanical cues within the adipose microenvironment have emerged as key regulators of progenitor fate and adipose tissue expandability. ECM remodeling and fibrosis increase matrix stiffness during obesity, activating integrin–focal adhesion kinase (FAK) signaling and downstream Hippo pathways mechanosensors that suppress adipogenic differentiation. Increased ECM stiffness promotes nuclear translocation of Yes-associated protein (YAP) and transcriptional coactivator with PDZ-binding motif (TAZ), which suppresses PPARγ activity, impairs adipogenic differentiation, and maintains progenitors in a fibro‑inflammatory state, thereby shifting adipose expansion toward hypertrophy rather than hyperplasia. Conversely, compliant ECM environments favor cytoplasmic retention of YAP/TAZ, enabling adipogenic commitment and supporting adaptive adipose tissue remodeling. These findings identify mechanotransduction and ECM-dependent signaling as critical regulators of adipose tissue plasticity and obesity-associated dysfunction [49, 50].

Taken together, anti–adipogenic pathways are a multilayered regulatory network that acts at distinct checkpoints of adipogenesis. AMPK restricts the metabolic shift required for differentiation, inflammatory cascades destabilize the adipogenic transcriptional machinery, TGF–β and Wnt signaling prevent lineage commitment and transcriptional progression, and sustained MAPK activation inhibits terminal maturation. These mechanisms ensure that adipogenesis proceeds only under favorable metabolic, hormonal, and environmental conditions.

Although signaling pathways determine whether adipogenesis is initiated or repressed, the execution of the differentiation process ultimately depends on a hierarchical transcriptional network that converts extracellular cues into stable adipocyte phenotype. Once progenitor cells receive appropriate hormonal and environmental signals, a tightly coordinated transcriptional cascade is activated, driving pre–adipocytes through early differentiation and toward a mature adipocyte phenotype.

### Adipogenesis Core Transcription Cascade

Adipogenesis is driven by a highly ordered and tightly regulated transcriptional cascade that converts external hormonal, nutritional, and metabolic cues into a stable adipocyte–specific gene expression program. This core transcriptional machinery operates downstream of lineage commitment and upstream of terminal phenotypic differentiation, acting as a nuclear regulatory framework that irreversibly establishes adipocyte identity. In parallel, adipogenesis involves profound changes in cell morphology, insulin sensitivity, and the reprogramming of the secretory profile that defines the endocrine functions of mature adipocytes [19].

In mammalian cells, adipogenesis is primarily controlled by the sequential activation of C/EBP transcription factors, followed by the induction of PPARγ, which acts as the central regulator of adipocyte lineage determination [21]. Importantly, the activity of these transcriptional regulators is tightly modulated by the PI3K–Akt–mTOR signaling axis, which integrates insulin, lipid synthesis and transport, growth factor, and nutrient signals, whose coordinated activity ensures the proper initiation, amplification, and stabilization of adipocyte differentiation [19].

#### Early Transcriptional Initiation: C/EBPβ and C/EBPδ

C/EBP belongs to a family of conserved basic leucine zipper transcription factors comprising six distinct isoforms, among which C/EBPβ, C/EBPδ and C/EBPα play the most prominent roles in driving adipocyte differentiation [51].

The earliest phase of adipogenesis is marked by the rapid and transient induction of the transcription factors C/EBPβ and C/EBPδ, which act as the primary molecular links between extracellular adipogenic stimuli and the transcriptional events that commit cells to the adipocyte differentiation program [52].

In classical in vitro models such as 3T3–L1 pre–adipocytes, the expression of C/EBPβ and C/EBPδ is induced within the first hours of differentiation following hormonal stimulation, particularly in response to insulin (which promotes glucose uptake and subsequent TGs storage), glucocorticoids such as dexamethasone (which preferentially induces C/EBPδ expression), and agents that elevate intracellular cAMP levels (such as 3–isobutyl–1–methylxanthine, IBMX, which primarily induces C/EBPβ) [53–55].

This hormonal cocktail activates the adipogenic program, leading to an early transcriptional response that precedes the activation of PPARγ and marks the first decisive molecular transition toward adipogenic commitment and subsequent differentiation. In vitro studies have shown that the loss of C/EBPβ and C/EBPδ significantly disrupts adipogenesis, inhibiting the proper induction of downstream regulators such as C/EBPα, PPARγ, and fatty–acid binding protein 4 (FABP4). Notably, in vivo studies have demonstrated that although adipogenesis can be initiated in the absence of C/EBPβ and C/EBPδ, adipose tissue development is severely compromised, indicating that adipocyte differentiation follows a tightly ordered transcriptional hierarchy in which the expression of C/EBPα and PPARγ, despite being essential, cannot independently support the formation of fully functional mature adipocytes [56].

Once induced, C/EBPβ and C/EBPδ bind to regulatory regions of multiple adipogenic genes, most notably those encoding PPARγ and C/EBPα, thereby initiating the transcriptional cascade that ultimately drives terminal adipocyte differentiation. Although both factors are expressed at this early stage, C/EBPβ plays a predominant role, whereas C/EBPδ functions mainly as a cooperative regulator that reinforces the adipogenic signal. Importantly, the transcriptional activity of C/EBPβ is not immediate, as it requires sequential post–translational modifications, including phosphorylation by MAPK and glycogen synthase kinase (GSK)3β, which are essential for its full DNA–binding capacity and transactivation potential [37, 57]. This temporal regulation ensures that cell–cycle progression during mitotic clonal expansion is properly coordinated with the onset of differentiation.

In addition to activating the core adipogenic regulators, C/EBPβ and C/EBPδ control a set of early–response genes involved in cell–cycle regulation, chromatin remodeling, and the initial metabolic adaptation of differentiating cells [58]. During mitotic clonal expansion, C/EBPβ supports the expression of cyclins and other proliferation–associated factors while simultaneously priming the chromatin landscape for subsequent PPARγ–driven transcription. Through this dual function, C/EBPβ serves as a key integrator of proliferative signals and differentiation cues during the early stages of adipogenesis [11, 33].

The expression, activity, and stability of C/EBPβ and C/EBPδ are tightly regulated by upstream signaling pathways, particularly the PI3K/Akt, MAPK/ERK, and cAMP/PKA cascades, which integrate hormonal, nutritional, and energy–related signals. Perturbations in these pathways markedly affect early adipogenic commitment by altering C/EBPβ and C/EBPδ induction or activation, thereby compromising PPARγ expression and subsequent adipocyte formation [11, 59]. Together, these early transcription factors define the entry point of the adipogenic program and play a decisive role in determining both the timing and efficiency of adipocyte differentiation.

#### Adipocyte Identity: PPARγ as the Master Regulator

The establishment of adipocyte identity is critically dependent on PPARγ, which functions as the central regulator of the adipogenic transcriptional program. Following the early induction of C/EBPβ and C/EBPδ, the activation of PPARγ marks the transition from a permissive differentiation state to a stable and irreversible mature adipocyte phenotype. At this stage, differentiation becomes self-sustaining, as PPARγ initiates a robust transcriptional network that consolidates both the morphological and metabolic features of mature adipocytes [59].

PPARs are ligand–dependent transcription factors of the nuclear hormone receptor family that regulate gene expression by binding to specific DNA response elements in target genes. Within the PPAR family, three main isoforms have been identified, PPARα, PPARβ (also known as PPARδ), and PPARγ, which display distinct tissue distributions and complementary metabolic functions. PPARα is highly expressed in metabolically active tissues such as the liver, heart, and brown adipose tissue, where it promotes fatty acid uptake and oxidation, ketogenesis, and metabolic adaptation to fasting. PPARβ/δ is ubiquitously expressed and acts as a key regulator of metabolic flexibility, coordinating lipid and glucose utilization, mitochondrial function, and oxidative metabolism across multiple tissues, including skeletal muscle and adipose tissue. In addition to its metabolic role, PPARβ/δ exerts anti-inflammatory and vasoprotective effects, linking energy metabolism to immunometabolic and cardiovascular homeostasis [60]. Among these isoforms, PPARγ plays a dominant and specialized role in adipogenic differentiation, glucose metabolism, and inflammatory regulation, establishing this receptor as a major pharmacological target for anti–diabetic and anti–obesity therapies [61, 62].

PPARγ is predominantly expressed in adipose tissue and functions as a master regulator of adipogenesis, driving adipocyte differentiation, lipid storage capacity, insulin sensitivity, and adipose tissue remodeling. Through the integrated actions of these isoforms, PPAR signaling coordinates systemic energy balance while shaping adipose tissue function and metabolic health [63]. PPARγ is expressed as two major protein isoforms, PPARγ_1_ and the adipocyte–specific PPARγ_2_, which arise from alternative promoter usage and differential splicing. Although PPARγ_1_ is broadly expressed across different tissues, PPARγ_2_ contains an additional N–terminal domain that confers enhanced adipogenic potency and is largely restricted to adipose tissue. Despite increased expression of both isoforms during adipocyte differentiation, PPARγ_2_ exhibits higher adipogenic activity and is considered the most functionally relevant isoform in adipose tissue. This preferential induction of PPARγ_2_ closely parallels the lipid droplet accumulation and acquisition of the metabolic features that characterize fully differentiated adipocytes [64].

Once expressed, PPARγ functions as a ligand–activated nuclear receptor that heterodimerizes with retinoid X receptor (RXR) and binds to peroxisome proliferator response elements within the promoters of adipocyte–specific genes [65]. Through this mechanism, PPARγ directly controls the expression of key regulators of lipid uptake, trafficking, and storage, including genes involved in fatty acid transport, TGs synthesis, glucose handling, and adipokine production, promoting the formation of small and insulin–sensitive adipocytes [66]. Canonical PPARγ target genes and lipid droplet–associated proteins such as FABP4, lipoprotein lipase (LPL), CD36, and glucose transporter (GLUT)4, collectively establish the lipid storage capacity of mature adipocytes. In parallel, PPARγ governs the expression of major adipokines, including adiponectin and leptin, linking intracellular lipid metabolism to the endocrine function of adipose tissue [62].

The transcriptional activity of PPARγ is subject to multilayered regulation involving ligand availability, post–translational modifications, and interactions with co–activators and co–repressors. Endogenous lipid–derived ligands promote PPARγ activation under physiological conditions, whereas synthetic agonists such as thiazolidinediones further enhance insulin sensitivity and glucose uptake [66]. Conversely, phosphorylation by kinases including ERK attenuates PPARγ activity and reshapes downstream metabolic responses, particularly those related to insulin sensitivity [62]. Through these regulatory mechanisms, PPARγ integrates hormonal, nutritional, and intracellular signaling cues to fine–tune adipocyte gene expression and functional adaptability.

Beyond its essential role in driving terminal differentiation, PPARγ is also required for the long–term maintenance of adipocyte identity and metabolic homeostasis. Disruption of PPARγ signaling in mature adipocytes leads to impaired lipid storage, increased ectopic lipid accumulation, and severe insulin resistance, underscoring its central role in whole–body energy balance [62]. Moreover, alterations in PPARγ expression or function contribute directly to adipose tissue dysfunction in obesity and related metabolic disorders [58]. Taken together, PPARγ operates as both the defining determinant of adipocyte identity and a key integrator of adipose tissue metabolism, plasticity, and systemic energy homeostasis.

#### Stabilization of Terminal Differentiation: C/EBPα

The stabilization of terminal adipocyte differentiation is critically dependent on C/EBPα, which functions as a key factor in locking in the mature adipocyte phenotype. Following the early induction of C/EBPβ and C/EBPδ and subsequent activation of PPARγ, C/EBPα expression is robustly upregulated at later stages of differentiation. At this point, C/EBPα no longer operates as an initiator of commitment but rather as a stabilizer of the fully differentiated state, reinforcing the transcriptional landscape that defines mature adipocytes [62].

C/EBPα cooperates closely with PPARγ in a positive feedback loop that is essential for maintaining high and sustained expression of adipocyte–specific genes. Studies have shown that each transcription factor can induce the other, indicating that, during the final stages of differentiation, C/EBPα and PPARγ act in a mutually reinforcing manner to drive the transcriptional program defining the mature adipocyte phenotype [56]. Both factors co–occupy numerous adipogenic targets, including genes involved in lipid uptake, TGs synthesis, lipid droplet formation, and insulin signaling [67]. Through this cooperative regulation, C/EBPα reinforces adipocyte metabolic specialization and ensures persistent activity of the adipogenic transcriptional network [59].

Once induced, C/EBPα drives the transition into terminal adipocyte differentiation by repressing proliferative gene expression and enforcing irreversible cell–cycle exit. In addition to consolidating the transcriptional network initiated by early adipogenic regulators, C/EBPα also controls the expression of essential metabolic genes that support adipocyte functionality, ensuring that structural differentiation is coupled to metabolic competence [52].

A key function of C/EBPα in mature adipocytes is the maintenance of insulin responsiveness. C/EBPα directly regulates the expression of critical insulin–signaling components, including the insulin receptor and GLUT4, enabling efficient glucose uptake and tight coupling of lipid storage to systemic glucose homeostasis [53]. Loss–of–function studies demonstrate that adipocytes lacking C/EBPα can still acquire certain morphological features of differentiation but exhibit severe defects in insulin sensitivity, highlighting the essential role of C/EBPα not only in establishing adipocytes structure but also in ensuring their full metabolic functionality [59].

After differentiation, C/EBPα expression remains high and is necessary to preserve adipocyte identity over time. Post–differentiation deletion of C/EBPα results in partial dedifferentiation, impaired lipid handling, and increased cellular plasticity, demonstrating that continuous C/EBPα activity is required for metabolic stability. Although PPARγ is necessary and sufficient to initiate adipogenesis and can stimulate lipid accumulation even in the absence of C/EBPα, adipocytes formed under these conditions display clearly reduced insulin sensitivity. This functional impairment illustrates that C/EBPα provides essential metabolic functions that PPARγ alone cannot fully compensate [53, 59]. Together, C/EBPα and PPARγ form a cooperative transcriptional module that stabilizes terminal differentiation and ensures that mature adipocytes remain metabolically competent.

#### Amplification and Late Functional Markers

The final stages of adipocyte differentiation are characterized by a broad amplification of the adipogenic transcriptional program and the robust expression of late functional markers that define the fully mature adipocyte phenotype. Once the core regulatory network driven by PPARγ and C/EBPα is firmly established, a large set of downstream genes involved in lipid metabolism, insulin responsiveness, and endocrine signaling is progressively upregulated. This amplification phase reflects not only quantitative increases in gene expression but also the functional maturation of adipocytes as specialized cells for energy storage and metabolic regulation.

During the late stages of adipocyte differentiation, metabolic specialization becomes increasingly pronounced, marked by the induction of key enzymes involved in lipid handling and storage. Among the most prominent functional markers of late adipocyte differentiation are fatty acid synthase (FAS), acetyl–CoA carboxylase (ACC), LPL, and FABP4, whose expression reaches maximal levels as adipocytes acquire the capacity for efficient fatty acid uptake, de novo synthesis, and TGs packaging. De novo lipogenesis is particularly enhanced at this stage, driven by the coordinated activation of ACC, FAS, and stearoyl–CoA desaturase 1 (SCD1), ensuring a sustained intracellular supply of fatty acids for TGs assembly and lipid droplet expansion [68]. FAS plays a central anabolic role by converting acetyl–CoA and malonyl–CoA into long–chain saturated fatty acids, which subsequently serve as essential substrates for TGs synthesis within the endoplasmic reticulum. This lipogenic flux is tightly coupled with insulin signaling and glucose uptake, linking carbohydrate availability to lipid storage capacity. The sustained activity of these enzymes, particularly FAS, is therefore crucial for the progressive enlargement and maturation of lipid droplets that characterize fully differentiated white adipocytes [69].

In parallel, lipid droplet–associated proteins such as perilipins are induced, ensuring not only the structural organization of lipid droplets but also their dynamic protection against uncontrolled lipolysis in response to hormonal cues [70]. At the same time, mature adipocytes acquire a fully functional lipolytic machinery through the coordinated induction of adipose triacylglycerol lipase (ATGL) and hormone sensitive lipase (HSL). ATGL catalyzes the initial and rate–limiting step of TGs hydrolysis, while HSL further processes diacylglycerols in response to catecholamines and other hormonal signals. The balanced activity of these enzymes allows mature adipocytes to dynamically shift between lipid storage during energy excess and lipid mobilization during fasting or increased energy demand, highlighting the metabolic plasticity of fully differentiated fat cells [71].

Terminal adipocyte differentiation is further characterized by the establishment of the adipocyte endocrine phenotype. As previously discussed, the expression and secretion of adipokines such as adiponectin, leptin, resistin, and a broad range of cytokines and chemokines become fully developed, allowing adipose tissue to communicate with distant organs including the liver, skeletal muscle, pancreas, and central nervous system. Through these signals, adipocyte metabolism is integrated into whole–body energy homeostasis. The precise balance of these adipokines is tightly linked to adipose tissue health, with alterations in their expression contributing directly to insulin resistance and low–grade chronic inflammation in obesity.

Simultaneously, the induction of the insulin–responsive GLUT4 enables efficient coupling between glucose uptake and lipogenesis, reinforcing the role of mature adipocytes as key regulators of systemic glucose homeostasis. In this integrated metabolic and secretory network, adipocytes actively participate in the fine–tuning of insulin sensitivity, appetite regulation, inflammatory state, and whole–body energy balance [72].

**Fig. 2:**
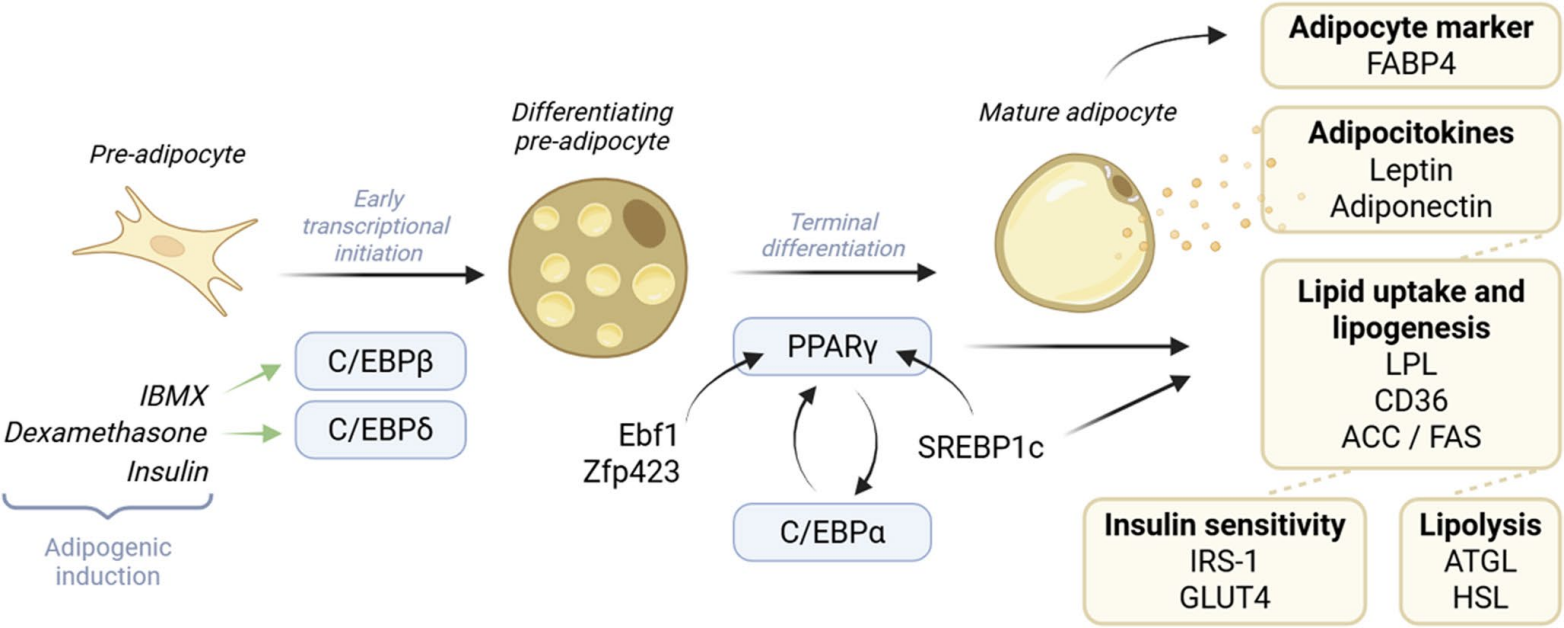
Transcriptional control of adipocyte differentiation and function. Adipogenic induction with isobutyl methylxanthine (IBMX), dexamethasone, and insulin activates CCAAT/enhancer-binding protein (C/EBP)β and C/EBPδ, promoting peroxisome proliferator-activated receptor (PPAR)γ expression. Early adipogenic competence is supported by early B-cell factor (Ebf)1 and zinc finger protein (Zfp)423, while PPARγ establishes a positive feedback loop with C/EBPα and cooperates with sterol regulatory element-binding protein (SREBP)1c to drive lipid uptake and de novo lipogenesis. Mature adipocytes secrete adipocytokines, including leptin and adiponectin, and exhibit enhanced lipid uptake and lipogenesis mediated by lipoprotein lipase (LPL), cluster of differentiation (CD)36, acetyl-CoA carboxylase (ACC), and fatty acid synthase (FAS), together with active lipolysis regulated by adipose triglyceride lipase (ATGL) and hormone-sensitive lipase (HSL). Insulin sensitivity is maintained by insulin receptor substrate (IRS)-1 and glucose transporter (GLUT)4, while fatty acid-binding protein 4 (FABP4) serves as a marker of mature adipocytes. Created in https://BioRender.com

Together, the progressive activation of lipid metabolic pathways, the acquisition of lipolytic competence, and the establishment of the adipokine secretory program (summarized in Fig. 2) mark the full functional maturation of adipocytes. At this stage, adipocytes are no longer merely differentiated cells but highly specialized metabolic and endocrine units that continuously adapt to nutritional status, hormonal signals, and energy demands. This integrated cellular and functional transition represents the physiological culmination of adipogenesis and reinforces the key role of adipose tissue in metabolic homeostasis and its dynamic adaptability in response to metabolic changes.

### Epigenetic and Post–Transcriptional Regulation

Adipocyte differentiation is not regulated exclusively by the ordered activation of transcription factors. Rather, it is profoundly influenced by epigenetic and post–transcriptional regulatory layers that determine the accessibility, timing, amplitude, and persistence of adipogenic gene expression. These mechanisms provide the molecular adaptability required to convert nutritional, hormonal, and mechanical cues into stable transcriptional programs that shape adipocyte identity. Through chromatin remodeling, DNA and histone modifications, the action of non–coding RNAs, and the selective control of mRNA processing and stability, pre–adipocytes progressively acquire both the competence to differentiate and the ability to maintain long–term metabolic function and adaptability [73].

At the chromatin level, adipogenesis involves a large–scale reorganization of chromatin structure, enabling the transition from a transcriptionally restricted progenitor state to a permissive adipocyte–specific gene expression landscape [74]. In undifferentiated pre–adipocytes, regulatory regions of key adipogenic genes, including PPARγ, C/EBPα, and FABP4, are maintained in a condensed and inactive configuration through repressive histone marks, most prominently histone H3 lysine 27 trimethylation (H3K27me3) and H3K9me3. Upon adipogenic induction, these inhibitory marks are gradually decreased, while permissive modifications such as H3K4me3, H3K27 acetylation (H3K27ac) and H3K9ac accumulate at promoters and enhancers, creating a chromatin environment that supports active transcription [75, 76].

The transition from a silent to an active adipogenic chromatin state is driven by a tightly coordinated interplay between histone–modifying enzymes. During the early stages of differentiation, histone acetyltransferases, particularly p300 and CREB–binding protein (CBP) are quickly recruited to adipogenic promoters downstream of early transcriptional regulators such as C/EBPβ and C/EBPδ, thereby enhancing chromatin accessibility and facilitating the activation of PPARγ and C/EBPα. In addition, histone deacetylases, which contribute to transcriptional repression in the undifferentiated state, are selectively displaced or inhibited as differentiation proceeds [76].

At enhancer regions, this process is preceded and reinforced by the activity of specific histone methyltransferases. The mono– and demethylation of H3K4 (H3K4me1 and H3K4me2) by mixed lineage leukemia (MLL)3 and MLL4 establishes a primed enhancer state that defines adipocyte–specific regulatory elements prior to full transcriptional activation. These primed enhancers subsequently acquire acetylation marks through CBP/p300 activity, leading to their functional activation and shaping a dynamic enhancer landscape that evolves throughout differentiation [75]. Concurrently, removal of repressive histone marks further consolidates this transition, as demethylases such as Jumonji domain–containing protein 3 (JMJD3, also known as KDM6B) erase H3K27me3, reinforcing enhancer activation in a stage–dependent manner [77]. Together, the sequential actions of MLL3/MLL4–mediated enhancer priming and CBP/p300–driven enhancer activation orchestrate the epigenomic reprogramming required for terminal adipocyte differentiation and plasticity [75].

DNA methylation provides another essential and highly dynamic layer of epigenetic control. In pre–adipocytes, CpG–rich regions within adipogenic promoters are typically hypermethylated, limiting transcriptional competence [78]. During differentiation, selective demethylation occurs at regulatory regions of genes such as PPARγ, adiponectin, and LPL, together with their transcriptional activation. These changes reflect both passive demethylation during clonal expansion and active processes mediated by ten–eleven translocation (TET) dioxygenases. Perturbations in DNA methylation patterns at adipogenic loci have been linked to impaired adipocyte differentiation, adipose tissue dysfunction, and metabolic disturbances in obesity, highlighting the importance of methylation dynamics in adipose plasticity [79].

Beyond chromatin–based mechanisms, non–coding RNAs constitute a highly responsive post–transcriptional regulatory network that shapes adipocyte differentiation. Among these, miRNAs are the most extensively characterized regulators in this context, acting mainly through mRNA degradation or translational repression. Many miRNAs display tightly regulated, stage–dependent expression patterns during adipocyte differentiation and directly target critical components of the adipogenic transcriptional cascade [76]. For example, miR–27 as well as miR–130 inhibit the master regulators PPARγ and C/EBPα, functioning as potent anti–adipogenic signals that preserve the pre–adipocyte state [80, 81]. In contrast, miR–143 is induced during differentiation and promotes adipocyte maturation, in part by modulating ERK5–PPARγ signaling [82]. Other miRNAs, including miR–21, miR–24, miR–30, miR–224, and miR–375, contribute to the regulation of insulin sensitivity, lipid accumulation, and mitochondrial metabolism in mature adipocytes [83].

Long non–coding RNAs (lncRNAs) further expand this regulatory complexity by acting as molecular scaffolds that coordinate chromatin remodeling, transcription factor recruitment, and post–transcriptional control [76]. Several adipogenesis–associated lncRNAs exhibit tightly controlled temporal expression profiles and exert either pro– or anti–adipogenic functions. A well–characterized example is adipogenic differentiation inducing noncoding RNA (ADINR), which promotes adipocyte differentiation by recruiting MLL3/4 complexes to the CEBPα promoter, thereby enhancing local H3K4 methylation and transcriptional activation. Other lncRNAs modulate adipogenesis through interactions with miRNAs or transcription factors, enabling context–dependent fine-tuning of adipogenic signaling [84].

Post–transcriptional gene regulation is further refined by RNA–binding proteins, which influence mRNA splicing, stability, transport, and translation. Proteins such as human antigen R (HuR), IGF2 mRNA–binding protein 2 (IGF2BP2) and members of the cytoplasmic polyadenylation element binding (CPEB) family dynamically associate with adipogenic transcripts to align gene expression with the metabolic and differentiation stage of the cell. By stabilizing mRNAs encoding key lipogenic enzymes, these proteins contribute to the establishment of the adipocyte metabolic phenotype. Moreover, alternative splicing events regulated by RNA–binding proteins generate adipocyte–specific protein isoforms with distinct functional properties [85].

Post–translational regulation provides yet another layer of integration between epigenetic, post–transcriptional and signaling mechanisms. Key adipogenic transcription factors such as PPARγ and C/EBPα undergo extensive phosphorylation, acetylation, ubiquitination, and sumoylation, which affect their stability, intracellular localization, and transcriptional activity [86]. These modifications respond dynamically to insulin signaling, nutrient availability, and inflammatory stimuli, allowing adipocytes to continuously adjust their transcriptional output in response to fluctuating metabolic demands [87].

Together, these epigenetic and post–transcriptional mechanisms establish a multilayered regulatory framework that confers both robustness and plasticity to the adipogenic program. While the core transcriptional cascade defines lineage identity, these additional regulatory layers determine the precision, durability, and adaptive capacity of adipocyte function. Dysregulation of epigenetic enzymes, non–coding RNAs, and RNA–binding proteins has been linked to adipose tissue dysfunction, insulin resistance, and obesity–associated metabolic diseases, highlighting their emerging relevance as potential therapeutic targets.

### Therapeutic Perspectives Targeting Adipogenesis

Advances in understanding the molecular and cellular mechanisms underlying adipogenesis and adipose tissue plasticity have significantly reshaped current therapeutic strategies for obesity and related metabolic disorders. Restoring adipose tissue plasticity and reversing dysfunctional adipogenesis require therapeutic strategies that directly or indirectly modulate the molecular pathways governing adipocyte commitment, differentiation, inflammation, metabolism, and mechanotransduction.

Rather than focusing exclusively on adipose tissue as a single target, current pharmacological approaches and emerging potential interventions increasingly aim to integrate multi-target frameworks on specific transcriptional and signaling pathways described in this review. Several of the pharmacological agents currently used for obesity were originally developed for other indications and later repurposed after their effects on metabolic and adipose–related pathways became evident, further illustrating the mechanistic convergence between systemic metabolic regulation and adipose tissue remodeling. This shift reflects the recognition that adipose tissue dysfunction arises from disruptions in the same adipogenic, inflammatory, metabolic, and mechanical pathways that regulate progenitor fate and depot-specific remodeling.

In this context, clinically approved anti–obesity drugs illustrate this mechanistic diversity. Orlistat reduces intestinal lipid absorption, indirectly limiting adipocyte hypertrophy, while incretin-based therapies such as liraglutide, semaglutide, and the dual glucose-dependent insulinotropic polypeptide (GIP)/GLP–1 receptor agonist tirzepatide act primarily through the gut-brain axis to suppress appetite and improve insulin sensitivity, thereby reducing inflammatory and metabolic pressure that impair adipogenic competence [88–90].

On the other hand, centrally acting combination therapies such as phentermine/topiramate and naltrexone/bupropion modulate hypothalamic pathways that intersect with ERK and AMPK–related signaling, whereas melanocortin 4 receptor (MC4R) agonist setmelanotide (for rare genetic forms of obesity) restores melanocortin signaling in monogenic obesity, indirectly influencing adipose tissue metabolism and expansion [91]. These combination therapies exemplify how simultaneous modulation of complementary neuroendocrine and metabolic pathways can indirectly influence adipose tissue remodeling and adipogenic capacity.

Importantly, the therapeutic objective in obesity is not to indiscriminately suppress adipogenesis, but to restore healthy adipose tissue expandability and plasticity. Favoring adipocyte hyperplasia over pathological hypertrophy, particularly within subcutaneous depots, while simultaneously restraining inflammation, fibrosis, and ectopic lipid spillover, offers a more physiologically coherent strategy for preserving metabolic homeostasis and requires interventions that target the molecular mechanism discussed earlier, including chronic inflammation, ECM stiffening, impaired progenitor commitment, and dysregulated metabolic signaling [92]. Within this conceptual framework, interventions targeting adipogenesis, energy expenditure, inflammation, and endocrine signaling can be viewed as complementary components of an integrated therapeutic approach rather than as opposing strategies [4].

Therapeutic strategies have traditionally aimed to limit excessive adipogenesis and lipid accumulation within WAT through adipogenic transcription factors modulation. This approach is largely focused on modulators of PPARγ and C/EBPα, including thiazolidinediones (TZD), that reinforce the terminal differentiation program and promote hyperplastic expansion, counteracting hypertrophic, inflamed adipose depots. Conversely, activation of AMPK, a central anti–adipogenic and anti–lipogenic regulator, suppresses mTORC1, reduces lipogenesis, and enhances fatty acid oxidation, shifting adipocyte metabolism toward a more catabolic state. Although many AMPK‑targeting compounds remain in preclinical development, they demonstrate how manipulating this metabolic checkpoint can reshape adipose tissue remodeling [93–95].

Beyond single‑target synthetic agents, bioactive dietary compounds, particularly flavonoids and other polyphenols, have attracted interest as complementary modulators of multiple adipogenic pathways simultaneously by influencing PPARγ, C/EBPα, AMPK, and epigenetic regulators, while attenuating oxidative stress and low‑grade inflammation. Their pleiotropic actions align with the multifactorial nature of adipose tissue dysfunction, although challenges in bioavailability and tissue‑specific delivery highlight the need for optimized formulations and targeted delivery systems [96].

To enhance the selectivity and efficacy of such interventions, targeted drug delivery systems based on nanomaterials are being actively explored. Nanocarriers capable of directing browning agents, metabolic modulators, or bioactive compounds specifically to adipose tissue may maximize therapeutic benefit while minimizing systemic off–target effects [97, 98]. These technologies represent an important translational bridge between mechanistic insights, such as the roles of PRDM16, UCP1, FAK, and YAP/TAZ, and translational applications, maximizing therapeutic efficacy while minimizing systemic effects.

A major conceptual advance has been the therapeutic induction of thermogenic adipocytes through the browning of WAT. This strategy directly targets pathways previously described, including cAMP/PKA, PRDM16, and UCP1. β3-adrenergic agonists, BMP7 analogs, and ILC2-activating cytokines promote beige adipocyte recruitment and mitochondrial uncoupling, increasing whole-body energy expenditure and improving metabolic flexibility [99, 100]. Targeting this axis offers the opportunity to reprogram adipose tissue from an energy–storing to an energy–dissipating organ, increasing whole–body energy expenditure and improving metabolic flexibility.

Beyond classical transcriptional regulation, emerging therapeutic strategies increasingly targeting deeper layers of cellular control, including autophagy and non–coding RNA networks. Dysregulation of autophagic flux in adipose tissue contributes to adipocyte hypertrophy, inflammation, and insulin resistance, while miRNAs, including miR–27, miR–155, and miR–143, act as key post–transcriptional regulators of adipogenic differentiation, lipid metabolism, and adipokine secretion [99, 101]. Although still predominantly explored in experimental models, these regulatory systems represent promising future therapeutic targets within the broader framework of adipose tissue plasticity.

The recent clinical success of GLP–1 receptor agonists and the development of dual and triple incretin–based agonists targeting combinations of GLP–1 and GIP with glucagon or amylin have demonstrated even greater efficacy in promoting sustained weight loss, further illustrating the transition toward integrated, multi–organ metabolic therapies [102, 103]. These next–generation agents illustrate how the simultaneous modulation of complementary hormonal pathways can generate synergistic metabolic effects that extend beyond appetite suppression, indirectly influencing insulin action, lipid handling, and adipose tissue remodeling and expandability.

Lastly, the integration of adipose tissue biology into precision medicine frameworks is gaining increasing relevance. The identification of obesity–associated adipose tissue dysfunction through histological, molecular, and circulating biomarkers enables refined patient stratification and the development of more personalized therapeutic strategies [104]. In parallel, growing attention is being directed toward neural and endocrine crosstalk between the central nervous system and adipose tissue, as well as the modulation of adipokines such as leptin and adiponectin, as complementary routes to restore metabolic homeostasis [105]. To synthesize these mechanistic relationships, Table 1 provides an integrated overview linking each therapeutic strategy to its primary molecular target, adipose tissue effect, and stage of development.

**Table 1:** Mechanistic overview of therapeutic strategies targeting adipogenesis and adipose tissue plasticity

Molecular pathway	Therapeutic strategies (examples)	Primarily molecular mechanism	Adipose tissue effects
Lipid absorption / Nutrient handling	Lipase inhibition *(Orlistat)*	Inhibition of pancreatic lipase: ↓ Dietary fat absorption	↓ Lipid influx into adipocytes ↓ Hypertrophy Indirect improvement of adipose tissue function
GLP–1 signaling	GLP-1 receptor agonists *(Liraglutide / Semaglutide)*	GLP-1R activation: ↑ AMPK ↓ Appetite and food intake ↑ Insulin sensitivity	↓ Hypertrophy ↓ Inflammation ↑ Thermogenesis (indirect) Indirect improvement of adipogenic competence
GIP/GLP-1 dual pathway	GIP/GLP-1 dual receptor agonists *(Tirzepatide)*	Co-activation of GIPR and GLP-1R: Enhanced incretin effect	↓ Inflammation Improved adipose remodeling
MC4R/POMC axis	MC4R agonist *(Setmelanotide)*	MC4R activation in hypothalamus: Restoration of leptin–melanocortin signaling	Restores energy balance in monogenic obesity Improved adipose tissue function
AMPK–mTOR signaling	AMPK activators *(Metformin / AICAR / Resveratrol)*	AMPK activation: ↓ mTORC1 ↑ Fatty acid oxidation	↓ Lipogenesis ↓ Lipid accumulation ↑ Insulin sensitivity
Adipogenic transcription factors	Anti-adipogenic compounds *(TZDs / Flavonoids)*	Inhibition of PPARγ and/or C/EBPα: Block terminal adipocyte differentiation	↓ Adipocyte differentiation ↓ Lipid accumulation ↓ Adipose tissue expansion
Browning / thermogenic program	Browning agents *(β3-agonists / BMP7 analogs / ILC2-activating cytokines)*	PRDM16 activation: ↑ UCP1 ↑ Mitochondrial biogenesis	↑ Thermogenesis ↑ Energy expenditure
Epigenetic modulation	miRNA-based therapeutics: *(miR-27 / miR-34 / miR155)*	Post-transcriptional regulation of adipogenic genes	Normalization of adipogenesis: ↓ Inflammation ↑ Metabolic homeostasis
Targeted delivery systems	Nanocarriers *(Liposomes / Exosomes)*	Adipose-targeted delivery: ↑ Bioavailability	↑ Specificity ↓ Systemic side effects

5‑aminoimidazol‑4‑carboxamida ribonucleotide (AICAR), AMP‑activated protein kinase (AMPK), bone morphogenetic protein (BMP), CCAAT/enhancer‑binding protein (C/EBP), glucose‑dependent insulinotropic polypeptide (GIP), glucose‑dependent insulinotropic polypeptide receptor (GIPR), glucagon‑like peptide (GLP), glucagon‑like peptide 1 receptor (GLP-1R), innate lymphoid cells (ILC), melanocortin‑4 receptor (MC4R), mechanistic target of rapamycin complex (mTORC), pro‑opiomelanocortin (POMC), peroxisome proliferator‑activated receptor (PPAR), PR domain‑containing protein 16 (PRDM16), transforming growth factor (TGF), thiazolidinediones (TZD) and uncoupling protein 1 (UCP1)

Beyond strategies aimed at reducing energy intake or modulating adipogenesis, increasing energy expenditure through activation of BAT and induction of beige adipocytes has emerged as a complementary therapeutic approach.

In contrast to white adipose tissue, BAT and beige adipocytes contribute to metabolic homeostasis through their capacity for adaptive thermogenesis. This process is primarily mediated by UCP1, which dissipates the mitochondrial proton gradient as heat rather than coupling it to ATP synthesis, thereby increasing energy expenditure. Beyond thermogenesis, activated BAT and beige adipocytes enhance glucose uptake, fatty acid oxidation, and lipid clearance, contributing to improved insulin sensitivity and systemic metabolic health. Beige adipocytes can be recruited within white adipose depots in response to stimuli such as cold exposure, exercise, β-adrenergic activation, and specific endocrine factors, highlighting the plasticity of adipose tissue [106]. Given the remarkable plasticity of adipose tissue, interventions that promote BAT activity or recruit beige adipocytes within white adipose depots may complement conventional anti-obesity therapies and represent promising approaches to reduce obesity and obesity-associated metabolic dysfunction.

Taken together, these advances support a clear conceptual transition from the simplistic suppression of fat mass toward the qualitative reprogramming of adipose tissue function. Future therapeutic success will likely depend on rational combination strategies that integrate pharmacological agents, bioactive compounds, targeted delivery technologies, and personalized interventions aimed at restoring the metabolic flexibility and endocrine competence of adipose tissue rather than only reducing its volume.

## Conclusions

Adipogenesis and adipose tissue plasticity emerge as central determinants of adipose tissue expandability, functional integrity, and systemic metabolic health. Rather than passive processes, they represent tightly regulated and highly dynamic mechanisms that govern how adipose tissue adapts to nutritional and environmental challenges. The capacity to generate new, metabolically competent adipocytes and to remodel tissue architecture is therefore critical in determining whether adipose expansion remains physiologically adaptive or progresses toward dysfunction.

As discussed throughout this review, efficient adipogenesis contributes to healthy adipose tissue expansion by promoting hyperplastic growth, preserving insulin sensitivity, and preventing ectopic lipid deposition. In contrast, impaired adipogenic capacity, often associated with chronic inflammation, fibrosis, progenitor cell dysfunction, and epigenetic alterations, shifts tissue expansion toward hypertrophy and metabolic rigidity. These alterations are linked to hypoxia, immune cell infiltration, dysregulated adipokine secretion, and systemic metabolic disturbances.

The regulation of adipogenesis is orchestrated by a complex but coordinated interplay of transcriptional, signaling, and epigenetic mechanisms that collectively define adipocyte identity and function. Core regulators such as PPARγ and C/EBP family integrate hormonal, nutritional, and inflammatory cues to direct lineage commitment and terminal differentiation, while pathways including Wnt, BMP, and insulin/IGF–1 modulate progenitor cell fate and adipogenic efficiency. These processes are further refined by epigenetic and post–transcriptional layers, which confer both stability and adaptability to adipocyte differentiation programs and contribute to long–term metabolic outcomes. Understanding how these pathways interact to maintain or disrupt adipogenic capacity is essential for explaining depot–specific differences in adipose function and identifying molecular targets capable of restoring healthy adipose tissue remodeling.

Adipose tissue plasticity extends beyond adipocyte turnover to include phenotypic flexibility, particularly the recruitment and activation of thermogenic beige and brown adipocytes. Modulating these processes through environmental, endocrine, or pharmacological stimuli represents a promising strategy to enhance energy expenditure and improve metabolic health. However, inter–individual variability, depot specificity, and long–term safety considerations highlight the need for further mechanistic and translational research.

Despite significant advances, important aspects of adipose biology remain incompletely understood, including the determinants of depot–specific adipogenic capacity, the reversibility of obesity–induced epigenetic alterations, and the mechanisms linking adipose tissue remodeling to systemic metabolic regulation. Addressing these challenges will require integrative approaches combining single–cell and spatial omics technologies with longitudinal human studies to better capture the dynamic nature of adipose tissue biology.

Overall, targeting adipogenesis and its underlying molecular regulatory networks represents a promising and conceptually refined strategy for the treatment of obesity and its associated metabolic disorders. Shifting the therapeutic focus toward restoring healthy adipocyte formation, improving tissue remodeling, and enhancing metabolic flexibility may provide more precise and durable benefits than approaches solely aimed at reducing fat mass.

## Key References

- Iacobini, C., et al., Impaired remodeling of white adipose tissue in obesity and aging: from defective adipogenesis to adipose organ Ddysfunction, Cells 13(9),doi: https://doi.org/10.3390/cells13090763 (2024).
  - ○ This study provides a comprehensive mechanistic overview of how impaired adipose tissue remodeling, particularly defective adipogenesis, drives adipose tissue dysfunction in obesity.
- Soták, M., et al., Inflammation and resolution in obesity, Nature Reviews Endocrinology 21(1): 45-61, doi: https://doi.org/10.1038/s41574-024-01047-y (2025).
  - ○ This review highlights the central role of chronic inflammation and its resolution in obesity, emphasizing immune–metabolic interactions within adipose tissue.
- Kokkorakis, M., et al., Emerging pharmacotherapies for obesity: A systematic review, Pharmacological Reviews 77(1): 100002, doi:https://doi.org/10.1124/pharmrev.123.001045 (2025).
  - ○ This systematic review summarizes emerging pharmacotherapies for obesity, integrating metabolic, hormonal, and adipose tissue–targeted strategies.
- Masliukov, P.M., Changes of signaling pathways in hypothalamic neurons with aging, Current Issues in Molecular Biology 45(10): 8289-8308, doi:https://doi.org/10.3390/cimb45100523 (2023).
  - ○ This work contributes to understanding systemic regulation of energy balance through central signaling pathways.
- Jung, B.C., et al., TET3 plays a critical role in white adipose development and diet-induced remodeling, Cell Reports 42(10): 113196, doi:https://doi.org/10.1016/j.celrep.2023.113196 (2023).
  - ○ This work demonstrates the role of epigenetic regulation, particularly DNA demethylation via TET3, in adipose tissue development and remodeling and provides important insight into how epigenetic mechanisms influence adipogenesis.

## Data Availability

No datasets were generated or analysed during the current study.

## Abbreviations

ACC: Acetyl CoA carboxylase
ADINR: Adipogenic differentiation inducing noncoding RNA
Akt: Protein kinase B
AMPK: AMP–activated protein kinase
ATGL: Adipose triacylglycerol lipase
ATM: Adipose tissue macrophage
ATP: Adenosine triphosphate
BAT: Brown adipose tissue
BMP: Bone morphogenic protein
C/EBP: CCAAT/enhancer–binding protein
cAMP: Cyclic adenosine monophosphate
CBP: CREB–binding protein
CD: Cluster differentiation
CPEB: Cytoplasmic polyadenylation element binding
CREB: cAMP response element–binding protein
Ebf: Early B-cell factors
ECM: Extracellular matrix
ERK: Extracellular signal–regulated kinase
FABP4: Fatty–acid binding protein 4
FAK: Focal adhesion kinase
FAS: Fatty acid synthase
GIP: Glucose–dependent insulinotropic polypeptide
GLP: Glucagon–like peptide
GLUT: Glucose transporter
GSK: Glycogen synthase kinase
H3K27ac: H3K27 acetylation
H3K27me3: Histone H3 lysine 27 trimethylation
HOX: Homeobox
HSL: Hormone sensitive lipase
HuR: Human antigen R
IBMX: 3–Isobutyl–1–methylxanthine
IFN: Interferon
IGF: Insulin–like growth factor
IGF2BP2: Insulin–like growth factor 2 mRNA–binding protein 2
IL: Interleukin
ILC: Innate lymphoid cells
iNKT: Invariant natural killer T
IRS: Insulin receptor substrate
JMJD3: Jumonji domain–containing protein 3
JNK: c–Jun N–terminal kinase
lncRNAs: Long non–coding RNAs
LPL: Lipoprotein lipase
MAPK: Mitogen–activated protein kinase
MC4R: Melanocortin 4 receptor
MLL: Mixed lineage leukemia
MSC: Mesenchymal stem cells
mTORC: Mammalian target of rapamycin complex
Myf: Myogenic factor
PDGFR: Platelet–derived growth factor receptor
PI3K: Phosphoinositide 3–kinase
PIP3: Phosphatidylinositol (3,4,5)–trisphosphate
PKA: Protein kinase A
POMC: Pro–opiomelanocortin
PPAR: Peroxisome proliferator–activated receptor
PRDM16: PR domain–containing 16
RXR: Retinoid X receptor
SAT: Subcutaneous adipose tissue
SCD1: Stearoyl–CoA desaturase 1
SMAD: Suppressor of mother against decapentaplegic
SREBP: Sterol regulatory element–binding protein
SVF: Stromal vascular fraction
TAZ: Transcriptional coactivator with PDZ-binding motif
TBX: T–box
TET: Ten–eleven translocation
TGF: Transforming growth factor
TGs: Triacylglycerides
TNF: Tumor necrosis factor
Tregs: Regulatory T cells
UCP1: Uncoupling protein 1
VAT: Visceral adipose tissue
WAT: White adipose tissue
Wnt: Wingless–related integration site
YAP: Yes-associated protein
Zfp: Zinc finger protein
